# The Impact of Spiritual Well-Being on Multidimensional Perfectionism in University Students: A Nationwide Survey

**DOI:** 10.3390/ejihpe15100211

**Published:** 2025-10-15

**Authors:** Alessio Lo Cascio, Elena Sandri, Anna De Benedictis, Anna Marchetti, Giorgia Petrucci, Silvia Dsoke, Gianluca Pucciarelli, Rosaria Alvaro, Maria Grazia De Marinis, Michela Piredda

**Affiliations:** 1 Department of Biomedicine and Prevention, University of Rome “Tor Vergata”, Via Montpellier, 1, 00133 Rome, Italy; alessio.locascio@hotmail.it (A.L.C.); gianluca.pucciarelli@uniroma2.it (G.P.); rosaria.alvaro@uniroma2.it (R.A.); 2 Faculty of Medicine and Health Sciences, Catholic University of Valencia San Vicente Mártir, c/Quevedo, 2, 46001 Valencia, Spain; elena.sandri@ucv.es; 3 Research Unit Nursing Science, Department of Medicine and Surgery, Campus Bio-Medico di Roma University, Via Alvaro del Portillo, 21, 00128 Rome, Italy; a.debenedictis@unicampus.it (A.D.B.);; 4 Research Unit Nursing in Palliative Care, Department of Medicine and Surgery, Fondazione Policlinico Universitario Campus Bio-Medico, Via Alvaro del Portillo, 200, 00128 Rome, Italy; s.dsoke@policlinicocampus.it; 5 Research Unit of Orthopaedic and Trauma Surgery, Department of Medicine and Surgery, Campus Bio-Medico di Roma University, Via Alvaro del Portillo, 200, 00128 Rome, Italy

**Keywords:** perfectionism, spiritual well-being, surveys and questionnaires, university students

## Abstract

Background: Perfectionism is a multidimensional personality trait encompassing both adaptive and maladaptive aspects that strongly influence students’ psychological health. Spiritual well-being, defined by existential and religious components, has been suggested as a protective factor, yet its relationship with perfectionism remains underexplored in university populations. This study aimed to investigate these associations in a large nationwide sample of Italian undergraduates. Methods: A total of 2103 students from public and private universities across Northern, Central, and Southern Italy participated in an online cross-sectional survey. Validated instruments were used to assess multidimensional perfectionism and spiritual well-being. Results: Self-oriented perfectionism emerged as the most prevalent dimension, followed by other-oriented and socially prescribed perfectionism. Scores for existential well-being were higher than those for religious well-being. Existential well-being was negatively associated with socially prescribed perfectionism, suggesting a buffering role against maladaptive forms of striving. Religious well-being showed only a small positive association with perfectionism. Gender and age differences were also observed, with women and younger students reporting higher levels of perfectionism. Conclusions: Findings highlight existential well-being as a potential protective factor in academic contexts, supporting meaning-centered strategies to mitigate maladaptive perfectionism. Longitudinal and cross-cultural studies are warranted to clarify causal mechanisms and inform culturally sensitive educational and clinical practices.

## 1. Introduction

Perfectionism has become increasingly prominent in contemporary higher education, particularly among university students facing elevated academic and social expectations. It is characterized by excessively high performance standards, overly critical self-evaluations, and heightened concern over mistakes (Frost et al., 1990; Hewitt & Flett, 1991). Conceptualized as a multidimensional construct, perfectionism includes self-oriented, socially prescribed, and other-oriented forms, each associated with distinct psychological risks (Hewitt et al., 2017). Self-oriented perfectionism reflects an internal drive for excellence, whereas socially prescribed perfectionism, where individuals perceive external pressure to meet unrealistic standards, has been consistently linked to adverse outcomes such as anxiety, depression, and suicidal ideation (Limburg et al., 2017; Smith et al., 2018).

The development of perfectionism is shaped by peer pressure (Sahi, 2018), family dynamics, and parenting styles, including excessive parental ambition and expectations (Schruder et al., 2014), or parental anxiety (Affrunti & Woodruff-Borden, 2017; Domocus & Damian, 2018). Difficulties in coping with failure also contribute to perfectionistic tendencies (Van der Kaap-Deeder et al., 2016).

Over recent decades, evidence indicates a global increase in perfectionism, particularly among younger generations, driven by rising societal pressures, neoliberal performance values, and intensifying parental expectations (Curran & Hill, 2019, 2022). Within universities, competitive and performance-driven environments may further amplify perfectionism, as students face demanding workloads, rigorous assessment criteria, and uncertain career trajectories (Beiter et al., 2015). Italian university students exhibit perfectionistic profiles similar to their international peers, with strong links to psychological distress, maladaptive coping, and reduced academic satisfaction (Nosè et al., 2025; Loscalzo et al., 2019; Gambolò et al., 2025).

Although perfectionism is associated with disengagement, procrastination, and emotional exhaustion, it remains a complex and insufficiently understood phenomenon (Al-Garni et al., 2025). Recent research has underscored the need to examine both its manifestations and antecedents, particularly in relation to perceived academic stress and understudied protective factors (Fernández-García et al., 2023; Piredda et al., 2025).

Spiritual well-being has emerged as a significant existential resource that enhances emotional balance, resilience and life satisfaction, while protecting against stress, anxiety, and depression (Leung & Pong, 2021; Okan et al., 2025; Rudolph & Barnard, 2023). Unlike religiosity, which centers on formal belief systems and rituals, spiritual well-being encompasses a broader sense of meaning and purpose derived from personal, relational, and transcendent connections, including engagement with art and music (Al-Thani, 2025; Ryff, 2021).

Although the relationship between spiritual well-being and perfectionism remains underexplored, related constructs such as self-compassion have been shown to buffer maladaptive effects, with higher levels linked to reduced depressive symptoms and psychological distress, even among self-critical perfectionists (Kawamoto et al., 2023; Tobin & Dunkley, 2021). In academic contexts, self-compassion moderates the relationship between perfectionistic striving and mental health, reducing stress and promoting well-being (With et al., 2024). Similarly, belonging to religious communities may mitigate the negative impact of socially prescribed perfectionism (Lin & Wang, 2024).

Broader evidence suggests that spirituality fosters life satisfaction, resilience, and lower anxiety, although causal mechanisms remain unclear (Benedetto et al., 2024; Rudolph & Barnard, 2023). A strong spiritual foundation may counterbalance perfectionistic tendencies by promoting intrinsic self-worth, self-compassion, and greater life satisfaction (Deb et al., 2020; Mathad et al., 2019), whereas low spiritual well-being may increase reliance on external validation, heightening vulnerability to maladaptive perfectionism (Negi et al., 2021). Moreover, the relational and transcendent aspects of spirituality may alleviate the interpersonal stressors inherent in socially prescribed perfectionism by fostering a broader perspective on human value and interconnectedness (Fisher et al., 2000).

Several studies have investigated perfectionism in Italian populations, including adolescents, young adults, and university students. For example, Di Fabio et al. (2019) explored its relationship with humor among university students, while Piredda et al. (2025) developed and validated the Perfectionism Inventory to assess perfectionism and academic stress. Other studies have linked perfectionism to eating behaviors (Vacca et al., 2021), procrastination and narcissistic vulnerability (Sommantico et al., 2024), and occupational outcomes such as work engagement and workaholism (Spagnoli et al., 2021).

Despite increasing attention to perfectionism among university students, the role of spiritual well-being remains largely unexplored. Previous studies have focused on related constructs such as self-compassion or religiosity, but few studies have examined how spiritual well-being may buffer or intensify perfectionistic tendencies. This gap is especially relevant in the Italian context, where spirituality blends cultural, personal, and religious dimensions that may shape students’ psychological functioning.

The present study investigates associations between spiritual well-being and multidimensional perfectionism in a large national sample of Italian university students. We hypothesized that (H1) higher spiritual well-being would relate negatively to maladaptive perfectionism and (H2) positively to adaptive perfectionism; and that (H3, exploratory) gender and age differences would emerge, with women and younger students reporting higher perfectionism.

## 2. Materials and Methods

### 2.1. Study Design

This study employed a multicentre, cross-sectional, observational design involving students enrolled at Italian universities.

### 2.2. Sampling and Data Collection

Participants were recruited through institutional mailing lists of universities in northern, central, and southern Italy, including the main islands, as well as through the investigators’ personal contacts. Eligibility criteria included enrolment in undergraduate or master’s degree programs at an Italian university and being at least 18 years old; students younger than 18 years were excluded. To maximize participation, two reminder emails were sent at one-week intervals following the initial invitation. Recruitment aimed to achieve broad representation of the Italian university population across geographic regions and academic levels.

Data were collected online between January 2024 and March 2025. All questionnaires were retained, as responses to essential items were mandatory, ensuring complete dataset. The study adhered to the STROBE guidelines for observational research (von Elm et al., 2007).

### 2.3. Instruments

*Multidimensional Perfectionism Scale—Revised (MPS-R).* The 14-item MPS-R was used to assess perfectionism (Piredda et al., 2025). It is a shortened version of the original 45-item MPS scale by Hewitt et al. (Hewitt et al., 2017; Hewitt & Flett, 1991). The MPS-R measures three dimensions of perfectionism: self-oriented perfectionism (SOP) through items 1, 3, 6, 9, and 12; other-oriented perfectionism (OOP) assessed via items 4, 7, 10, and 13; and socially prescribed perfectionism (SPP), measured by items 2, 5, 8, 11, and 14 (see Table S1 in Supplementary File). The MPS-R was validated by Piredda et al. (2025) through Confirmatory Factor Analysis yielding good fit indices (RMSEA = 0.048 (IC 90% 0.037–0.059), *p* = 0.594; CFI = 0.948; TLI = 0.935; SRMR = 0.049) and good reliability (Omega values for first-order factors, SOP, OOP, SPP: 0.77, 0.67, 0.79; global reliability index and Omega H for second-order factors: 0.73 and 0.60). Each item is rated on a 7-point Likert scale ranging from 1 (“strongly disagree”) to 7 (“strongly agree”). The overall score is computed by summing the responses to all items, yielding a total score between 14 and 98.

*Spiritual Well-Being Scale (SWBS).* Spiritual well-being was investigated through the validated and widely used 20-item SWBS (Moberg, 1979; Moberg & Brusek, 1978). It comprehensively assesses spiritual well-being, identifying both religious and existential dimensions. The scale includes ten items that focus on Religious Well-Being (RWB), explicitly referencing “God,” and another ten items that measure Existential Well-Being (EWB), which explore aspects such as meaning, satisfaction, and direction in life without invoking religious terminology. The items of SWBS are rated on a 6-point Likert scale ranging from “Strongly Disagree” (1) to “Strongly Agree” (6). Three primary scores were derived from the Spiritual Well-Being Scale (SWBS): Overall Spiritual Well-Being (SWB), Religious Well-Being (RWB), and Existential Well-Being (EWB). Internal consistency measured in several samples through Cronbach’s alpha coefficients ranged 0.82–0.94 (RWB), 0.78–0.86 (EWB), and 0.89–0.94 (SWB) (Bufford et al., 1991). Positively worded items (e.g., items 3, 4, 7, 8, 10, 11, 14, 15, 17, 19, 20) were scored directly, whereas negatively worded items (e.g., items 1, 2, 5, 6, 9, 12, 13, 16, 18) were reverse coded to ensure that higher scores consistently reflected greater spiritual well-being. The overall SWB score, ranging from 0 to 120, was calculated by summing responses to all 20 items. Scores between 20 and 40 were classified as indicating low spiritual well-being, scores between 41 and 99 as moderate, and scores between 100 and 120 as high spiritual well-being. The RWB and EWB subscale scores, each ranging from 0 to 60, were similarly categorized: low (10–20), moderate (21–49), and high (50–60) levels of religious or existential well-being, respectively.

**Figure 1:**
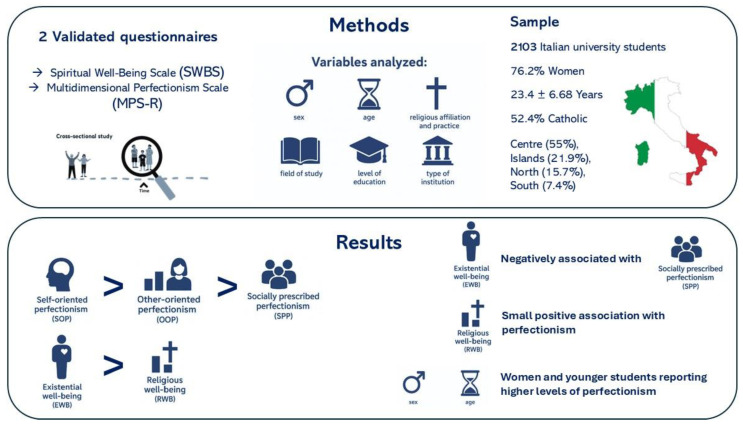
Graphic abstract of the research process and main results.

Socio-demographic and academic information was also collected, including sex, age, religious affiliation and practice, as well as academic context variables such as field of study, level of education, and type of institution (e.g., private university). Age was grouped into three categories: 18–24 years (young), 25–39 years (young adults), and 40–62 years (middle-aged). Figure 1 presents a graphic abstract of research process and main results.

### 2.4. Ethical Considerations

The study adhered to the ethical principles outlined in the Declaration of Helsinki (World Medical Association [WMA], 2025). The relevant Ethics Committees approved the study (Protocol FPUCBM 001.23[45.22] OSS, 19 April 2023, and 75.23CET2cbm, 26 October 2023). Participant confidentiality and data protection were ensured in compliance with current regulations. Informed consent for participation and data processing was obtained online prior to questionnaire completion.

### 2.5. Data Analysis

The dataset was pre-processed to remove invalid, inconsistent, or extreme entries, including inaccurate responses or statistical outliers. Following data cleaning, the Shapiro–Wilk test was employed to assess distribution normality, revealing that none of the variables were normally distributed. These results were visually corroborated using Q–Q plots (von Elm et al., 2007). Given the non-normal distribution, appropriate non-parametric statistical tests were applied: the Chi-square test for categorical variables, and the Mann–Whitney U or Kruskal–Wallis tests for comparisons between two or more groups, respectively. To account for multiple comparisons between groups, the Holm–Bonferroni adjustment was applied to control for Type I error. For the multivariate analysis, a multiple linear regression was employed, since this approach requires normality of residuals rather than predictors. Model assumptions were verified: residuals showed no autocorrelation (Durbin–Watson = 2.00, *p* = 0.898), VIF values (1.02–2.17) indicated no relevant multicollinearity, residuals displayed reasonable normality despite Shapiro–Wilk deviations, and plots confirmed homoscedasticity. This enabled simultaneous evaluation of the predictive contributions of perfectionism dimensions and key socio-demographic factors. All statistical analyses were performed using Jamovi software (Version 2.6.26). Additional visualizations and figure enhancements were created using Microsoft Excel.

## 3. Results

### 3.1. Sample Description

Table 1 summarizes the socio-demographic characteristics of the sample, which comprised 2103 university students. The gender distribution was notably uneven, with 76.2% identifying as females and 23.8% as males. The mean age was 23.4 years (SD = 5.68), with the majority aged 18–24 (76.46%). In terms of religious affiliation, 52.4% identified as Catholic. A combined 27.2% described themselves as Atheist or Agnostic, and 13.9% expressed indifference toward religion. Smaller portions reported affiliation with Orthodox Christianity (1.9%), other Christian denominations (1.9%), Islam (0.8%), and other faiths. Only 28.9% reported active religious practice. Regarding academic discipline, 70.2% were enrolled in health-related programs. Students in the humanities accounted for 13.1%, engineering for 8.3%, and science for 5.8%. Academic year distribution was relatively balanced, with the largest groups in the first (23.2%) and second years (41.9%). Regionally, most participants were from central Italy (55%), followed by the Islands (21.9%), and the North (15.7%). A majority attended public universities (81.6%), while 18.4% were enrolled in private institutions. Additionally, 35.8% were offsite students, and 19.4% reported receiving either a scholarship or placement in a College of Merit.

### 3.2. Analysis of MPS-R Responses

The Cronbach’s alpha coefficient for Multi-dimensional Perfectionism Scale–Revised (MPS-R) was 0.74. Table 2a–c summarizes responses to MPS-R across its three dimensions: self-oriented (SOP), other-oriented (OOP), and socially prescribed perfectionism (SPP).

Self-oriented perfectionism (SOP) was strongly endorsed, with participants reporting high personal standards, striving for excellence, and discomfort with mistakes. Items such as “I set very high standards for myself” and “It makes me uneasy to see an error in my work” received widespread agreement, indicating that self-directed perfectionism was pervasive.

Other-oriented perfectionism (OOP) showed moderate endorsement. Students held high expectations for important others, but these interpersonal standards were less prominent than their own self-directed standards.

Socially prescribed perfectionism (SPP) reflected moderate perceptions of external pressures. While some students felt that others expected them to succeed, these pressures were less internalized compared to their personal standards, suggesting that external demands were acknowledged but not strongly adopted.

Table 3 PART A summarize descriptive statistics for the MPS-R items and domains. Participants showed strong self-oriented perfectionism (SOP), with the highest scores on items reflecting discomfort with mistakes and high personal standards, highlighting an internal drive for flawlessness. Other-oriented (OOP) and socially prescribed perfectionism (SPP) were endorsed less strongly, indicating that perfectionistic tendencies were primarily self-imposed rather than externally driven. At the domain level, SOP had the highest mean, followed by OOP and SPP, and the overall MPS-R score indicated a moderate to high prevalence of perfectionistic traits.

### 3.3. Analysis of Spiritual Well-Being Scale (SWBS) Responses

Cronbach’s alpha coefficients were 0.970, 0.893 and 0.937 for RWB, EWB and SWB, respectively. The results in Table 3 PART B indicate that Italian university students report a moderate overall level of spiritual well-being (SWBS = 68.8). Within this construct, existential well-being (EWB) was notably higher than religious well-being (RWB), suggesting that participants derive greater meaning and satisfaction from internal resources than from religious beliefs or practices. Positive appraisals of life and purpose were strongly endorsed, while negative or nihilistic statements were largely rejected. In contrast, items assessing religious well-being indicated a weak personal connection with God, with low agreement on statements reflecting a meaningful relationship or perceived support from God. For further details of the comparison, Table A1 in Appendix A presents the results from Table 3 also divided by sex.

### 3.4. Associations Between Perfectionism and Spiritual Well-Being

The correlation matrix (Table 4) revealed distinct patterns between perfectionism dimensions (MPS-R items) and spiritual well-being variables.

Self-oriented perfectionism indicators (e.g., “It makes me uneasy to see an error in my work,” “I set very high standards for myself”) showed weak to moderate negative correlations with existential well-being items, such as life satisfaction, optimism about the future, and sense of purpose (r range ≈ −0.05 to −0.12, *p* < 0.05 to *p* < 0.001). These results suggest that higher self-imposed standards and intolerance of mistakes are associated with lower existential clarity and fulfillment.

Items reflecting socially prescribed perfectionism (e.g., “I find it difficult to meet others’ expectations of me,” “The people around me expect me to succeed at everything I do”) demonstrated the strongest negative associations with existential well-being, including life satisfaction and perceived purpose (r range ≈ −0.20 to −0.40, all *p* < 0.001). This indicates that perceived external demands are particularly detrimental to personal meaning and well-being.

By contrast, other-oriented perfectionism (e.g., “Everything that others do must be of top-notch quality,” “I cannot stand to see people close to me make mistakes”) showed weak positive correlations with certain religious well-being items, such as belief in God’s love or support (r ≈ 0.05 to 0.12, *p* < 0.05 to *p* < 0.001). However, these associations were smaller in magnitude compared to the negative links observed for self- and socially prescribed perfectionism.

Overall, the results highlight that perfectionism is differentially related to spiritual well-being: socially prescribed perfectionism exhibits the most consistent and detrimental correlations with existential dimensions, while self-oriented perfectionism is linked to reduced fulfillment and optimism, and other-oriented perfectionism displays only minor associations with religious beliefs.

### 3.5. Multiple Linear Regression Analysis Between Dimensions of Perfectionism, Spiritual Well-Being and Related Socio-Demographic Factors

The linear regression model (Table 5) examining the association between perfectionism (MPS-R) and the dimensions of spiritual well-being explained a modest proportion of the variance (R = 0.315, R^2^ = 0.099, N = 2103), indicating that these predictors accounted for approximately 10% of the variance in perfectionism.

Existential Well-Being (EWB), which reflects a relatively weak effect which reflects meaning, purpose, satisfaction, and perceived direction in life without invoking religious terminology, was the strongest predictor, showing a significant negative association with perfectionism (β = −0.369, SE = 0.028, t = −13.117, *p* < 0.001). Religious Well-Being (RWB), which explicitly refers to a personal relationship with God and the belief that God provides care and guidance, showed a small but significant positive effect (β = 0.067, SE = 0.024, t = 2.754, *p* = 0.006).

Sociodemographic covariates also contributed: women reported higher perfectionism than men (β = 1.662, SE = 0.597, t = 2.785, *p* = 0.005), while young adults (β = −2.825, SE = 0.645, t = −4.379, *p* < 0.001) and middle-aged participants (β = −3.929, SE = 1.491, t = −2.635, *p* = 0.008) exhibited lower perfectionism than the youngest group. Other demographic variables, including religious affiliation beyond Catholicism, area or level of study, geographic location, type of institution (public vs. private), off-site residence, and scholarship status, were not significant.

## 4. Discussion

This survey investigated the relationship between spiritual well-being, including both religious and existential dimensions, and multidimensional perfectionism, as well as the socio-demographic and contextual factors shaping these associations. Drawing on a large sample of 2103 Italian students, the study provides insights into how spiritual well-being interacts with perfectionism in higher education.

A moderate to high prevalence of perfectionism emerged, with self-oriented perfectionism (SOP) most prominent, followed by other-oriented (OOP), and socially prescribed perfectionism (SPP). This pattern underscores the predominance of an internalized drive for high standards, while external and interpersonal demands appear less influential. SOP’s prominence resonates with theoretical accounts emphasizing its dual role, as both a motivational asset and a vulnerability depending on the context (Hewitt et al., 2017; Hewitt & Flett, 1991; Stoeber & Otto, 2006). The absence of negative associations between SOP and existential well-being suggests that, in this population, SOP may reflect a form of adaptive striving for excellence, characterized by intrinsic motivation and goal pursuit, rather than maladaptive perfectionism. This interpretation is consistent with emerging work distinguishing healthy striving or “excellencism” from rigid, self-critical perfectionism (Gaudreau et al., 2022), though further research is required to substantiate this distinction. In contrast, OOP and SPP, typically linked with interpersonal conflict and psychological maladjustment (Hewitt & Flett, 1991), were less prevalent, suggesting a relatively low level of maladaptive striving in this sample.

Overall spiritual well-being was moderate, with existential well-being (EWB) consistently outweighing religious well-being (RWB). While formal religious engagement appears limited, students reported a strong sense of meaning, purpose, and optimism. This reflects a reliance on existential meaning-making over traditional religiosity, consistent with broader European trends of secularization and the search for meaning outside traditional religious institutions among youth (Bossi et al., 2023; Lo Cascio et al., 2025; Rudolph & Barnard, 2023).

Associations between spiritual well-being and perfectionism were significant but differentiated. Higher EWB was negatively related to SPP, highlighting a protective effect against maladaptive perfectionism, which is strongly linked with depression, anxiety, and suicidality (Limburg et al., 2017; Smith et al., 2018). These results support evidence that spirituality helps students frame striving within broader systems of meaning, mitigating risks of burnout (Mathad et al., 2019; With et al., 2024). RWB, by contrast, showed a small positive association with perfectionism. While the effect was weak, it may reflect that religious adherence sometimes reinforces external standards or evaluative concerns, even as it provides belonging and support. This dual role warrants further study.

Beyond these direct associations, the interplay between spirituality, perfectionism, and broader psychosocial mechanisms deserves deeper consideration. Perfectionism, particularly in its socially prescribed form, is embedded in relational and cultural contexts where external validation, competition, and performance pressures dominate. Spirituality, especially existential well-being, appears to counterbalance these dynamics by shifting the basis of self-worth from external evaluation to intrinsic meaning, belonging, and acceptance. This aligns with broader psychosocial theories that position spirituality as a resilience factor fostering social connectedness, emotional regulation, and purpose-driven coping (McEntee et al., 2013). In this framework, spirituality not only mitigates perfectionism’s maladaptive outcomes but also situates striving within a coherent life narrative, reducing vulnerability to anxiety and burnout. Conversely, when spiritual well-being is low, students may rely more heavily on achievement and external approval as sources of identity, reinforcing perfectionistic standards and their psychosocial costs. Thus, spirituality may function as a mediating mechanism linking perfectionism with broader psychosocial outcomes such as distress, interpersonal conflict, and academic disengagement. Future studies should explicitly model these mediating pathways to clarify how spiritual resources shape the social and psychological consequences of perfectionism.

Demographic differences were also observed. Women reported higher perfectionism than men, echoing prior findings linking gendered expectations and evaluative fears to perfectionistic tendencies (Ghosh & Roy, 2017). Younger students displayed higher levels of perfectionism compared with older peers, suggesting a developmental trend whereby perfectionistic tendencies may diminish with age as individuals gain broader life perspectives and self-acceptance. This difference may have been detected due to the large sample size, as prior studies with smaller cohorts yielded inconsistent results (Daniilidou, 2023; Diamantopoulou & Platsidou, 2014; Landa & Bybee, 2007; Robinson et al., 2021). The lack of significant differences across institutions, regions, or disciplines suggests that perfectionism and spiritual well-being and its existential correlates represent a widespread concern across the Italian university system.

These findings carry practical significance for higher education. Acknowledging the role of spiritual well-being in moderating perfectionism can inform the design of holistic student support initiatives.

First, routine screening for perfectionism and low existential well-being could be incorporated into university counseling services to identify at risk students early. Targeted support may include meaning-centered interventions, such as mindfulness, values clarification, narrative reflection, and peer-support groups. These approaches can help students reframe perfectionistic standards, cultivate self-acceptance and reduce reliance on external validation.

Second, universities can enhance campus environments that promote spiritual and existential development beyond formal religious practice. Opportunities such as service learning, volunteerism, intercultural dialogue, arts engagement arts, and access to natural spaces can strengthen belonging, purpose, and resilience. Multi-faith or chaplaincy services can also provide safe spaces for students to explore existential concerns and cultivate meaning in inclusive ways (Barton et al., 2020). For students who identify with a religious tradition, spiritual guidance may further reinforce self-worth and belonging. For example, the notion of a compassionate and forgiving God, central to monotheistic religions such as Christianity in the Italian context, can offer a counterbalance to the fear of failure and excessive reliance on external validation, thereby supporting more adaptive forms of striving (Duffield et al., 2024; Saliba, 2025).

Third, academic staff play a crucial role in shaping how perfectionism is experienced. Faculty training programs should equip educators to recognize perfectionistic tendencies and adopt pedagogical approaches that balance high standards with psychological safety. Evidence-based practices include formative assessments that emphasize progress over flawless performance, timely and constructive feedback, and explicit normalization of mistakes as part of learning (Brookhart, 2011; Fried, 2011). Such approaches can help mitigate performance pressure while maintaining motivation for excellence.

Finally, structural measures at the institutional level are needed. Universities should expand counseling services, develop psychoeducational workshops on perfectionism, and launch awareness campaigns to normalize help-seeking and challenge performance-driven cultures. Evidence supports the effectiveness of brief cognitive-behavioral programs and resilience-building workshops in reducing maladaptive perfectionism (Arana et al., 2017). Adapting these approaches for university settings can help reframe perfectionism not merely as an individual issue but as a systemic challenge within academic culture. By embedding meaning-centered practices within both counseling and teaching structures, universities can promote achievement while protecting student well-being.

Future research should pursue longitudinal and qualitative approaches to clarify causal mechanisms and subjective experiences. Comparative studies across cultural contexts would also illuminate whether the observed patterns reflect universal trends or specific features of Italian higher education. Moreover, differentiating between adaptive striving and maladaptive perfectionism in relation to existential well-being could refine our understanding of protective versus risk pathways.

### Strengths and Limitations

This nationwide survey, the largest to date on spiritual well-being and perfectionism in Italian universities, included over 2100 students across regions and disciplines. Its strengths lie in sample diversity, the use of validated instruments, and adherence to STROBE guidelines, all of which enhance reliability and validity.

Nevertheless, several limitations must be acknowledged. First, the cross-sectional design prevents causal inference. While higher existential well-being may reduce maladaptive perfectionism, it is equally possible that perfectionistic tendencies influence spiritual resources. Longitudinal research is needed to clarify directionality. Second, reliance on self-report introduces potential bias, including social desirability and distorted self-perceptions, particularly concerning sensitive constructs such as spirituality. Third, despite the nationwide scope, the sample composition was not fully representative: women and younger students were overrepresented, and a substantial proportion of respondents were enrolled in health-related programs. Although this gender and age distribution reflects national university demographics (AlmaLaurea, 2024), the imbalance limits the generalizability of findings, as perfectionism and spiritual well-being may vary across genders, age groups, and academic fields. Future studies should employ targeted recruitment strategies to ensure more balanced representation. Finally, the use of online recruitment may have attracted students already interested in perfectionism or spirituality. Randomized or stratified sampling approaches would enhance representativeness in future research.

## 5. Conclusions

This study suggests that existential well-being is a protective factor against socially prescribed perfectionism among university students. While perfectionism is deeply embedded in academic culture, fostering meaning, purpose, and intrinsic self-worth may help students navigate pressures without succumbing to maladaptive outcomes.

For universities, the findings translate into several actionable recommendations. Institutions should integrate meaning-centered approaches into counseling and student support services, expand co-curricular opportunities for existential and spiritual development, and promote learning environments where mistakes are normalized as integral to growth. Faculty development initiatives should prepare educators to recognize and address perfectionism, while systemic interventions—such as workshops, awareness campaigns, and resilience training—can reduce stigma, enhance coping resources, and promote psychological safety. Universities should also recognize the role of chaplaincy and multi-faith services in supporting students’ spiritual well-being. In contexts where monotheistic traditions are prominent, faith-based perspectives emphasizing compassion, forgiveness, and unconditional worth may provide additional resources to counteract maladaptive perfectionism and reinforce belonging and resilience

By addressing perfectionism as both an individual and cultural issue, universities can create inclusive and sustainable learning environments that support academic excellence alongside mental health. These findings underscore the value of integrating existential and spiritual perspectives into student well-being initiatives, with potential long-term benefits for both academic adjustment and overall psychological resilience.

## Figures and Tables

**Table 1:** Sample socio-demographic characteristics (N = 2103 students).

**Sex**	**N**	**%**
Male	500	23.80%
Female	1603	76.20%
**Age**	**Mean**	**SD**
	23.4	5.68
	**N**	**%**
Young (18–24 years)	1608	76.46%
Young adults (25–39 years)	431	20.45%
Middle age (40–62 years)	65	3.09%
**Religious Faith**	**N**	**%**
Roman catholic	1103	52.40%
Other religion	137	6.50%
Atheist/Agnostic	571	27.20%
Indifferent	292	13.90%
**Religious Practice**	**N**	**%**
No	1496	71.10%
Yes	607	28.90%
**Study Level**	**N**	**%**
Bachelor’s degree	1653	78.60%
Master’s degree	450	21.40%
**Course of Studies**	**N**	**%**
Health science (Nursing, Medicine, Dentistry, Nutrition, Psychology)	1477	70.20%
Humanities (Philosophy, Literature, Communication, Law, Educational sciences, Economics, etc.)	275	13.10%
Engineering	175	8.30%
Science (Mathematics, Physics, Chemistry, Biology, Biotechnology, etc.)	122	5.80%
Other	54	2.60%
**Course Year**	**N**	**%**
1	488	23.20%
2	881	41.90%
3	396	18.80%
4	120	5.70%
5	147	7.00%
6	71	3.40%
**Geographical Area**	**N**	**%**
North	330	15.70%
Centre	1157	55.00%
South	156	7.40%
Main islands	460	21.90%
**Type of University**	**N**	**%**
Public	1717	81.60%
Private	386	18.40%
**Offsite Student**	**N**	**%**
No	1145	64.18%
Yes	638	35.76%
No answer	320	17.94%
**Scholarship/Place in College of Merit**	**N**	**%**
No	1431	80.62%
Yes	344	19.38%
No answer	328	18.48%

Legend: SD = Standard Deviation.

**Table 2:** (a) Distribution of responses for the SOP dimension of the MPS-R scale. (b) Distribution of responses for the OOP dimension of the MPS-R scale. (c) Distribution of responses for the SPP dimension of the MPS-R scale.

(**a**)												
**SOP (Self-Oriented Perfectionism)**												
	**When I Am Working on Something, I Cannot Relax Until It Is Perfect**			**One of My Goals Is to Be Perfect in Everything I Do**		**It Makes Me Uneasy to See an Error in My Work**		**I Do not Have to Be the Best at Whatever I Am Doing**		**I Set Very High Standards for Myself**		
	**N**	**%**		**N**	**%**	**N**	**%**	**N**	**%**	**N**		**%**
1. Strongly disagree	30	1.40%		150	7.10%	24	1.10%	368	17.50%	54		2.60%
2. Disagree	65	3.10%		145	6.90%	29	1.40%	276	13.10%	45		2.10%
3. Somewhat disagree	162	7.70%		202	9.60%	68	3.20%	430	20.40%	108		5.10%
4. Neither agree nor disagree	266	12.60%		320	15.20%	144	6.80%	409	19.40%	218		10.40%
5. Somewhat agree	800	38.00%		591	28.10%	698	33.20%	318	15.10%	533		25.30%
6. Agree	444	21.10%		400	19.00%	511	24.30%	183	8.70%	474		22.50%
7. Strongly agree	336	16.00%		295	14.00%	629	29.90%	119	5.70%	671		31.90%
(**b**)												
**OOP (Other-Oriented Perfectionism)**												
	**Everything That Others Do Must Be of Top-Notch Quality**				**I Cannot Stand to See People Close to Me Make Mistakes**		**I Have High Expectations for the People Who Are Important to Me**		**The People Who Matter to Me Should Never Let Me Down**			
	**N**		**%**		**N**	**%**	**N**	**%**	**N**		**%**	
1. Strongly disagree	320		15.20%		417	19.80%	80	3.80%	81		3.90%	
2. Disagree	319		15.20%		364	17.30%	120	5.70%	124		5.90%	
3. Somewhat disagree	381		18.10%		350	16.60%	154	7.30%	190		9.00%	
4. Neither agree nor disagree	659		31.30%		502	23.90%	481	22.90%	346		16.50%	
5. Somewhat agree	285		13.60%		330	15.70%	726	34.50%	622		29.60%	
6. Agree	87		4.10%		96	4.60%	324	15.40%	355		16.90%	
7. Strongly agree	52		2.50%		44	2.10%	218	10.40%	385		18.30%	
(**c**)												
**SPP (Socially Prescribed Perfectionism)**												
	**I Find It Difficult to Meet Others’ Expectations of Me**			**I Feel That People Are Too Demanding of Me**		**The People Around Me Expect Me to Succeed at Everything I Do**		**My Family Expects Me to Be Perfect**		**Success Means That I Work Even Harder to Please Others**		
	**N**	**%**		**N**	**%**	**N**	**%**	**N**	**%**	**N**		**%**
1. Strongly disagree	170	8.10%		160	7.60%	136	6.50%	380	18.10%	650		30.90%
2. Disagree	260	12.40%		181	8.60%	151	7.20%	254	12.10%	252		12.00%
3. Somewhat disagree	266	12.60%		212	10.10%	210	10.00%	286	13.60%	363		17.30%
4. Neither agree nor disagree	464	22.10%		468	22.30%	433	20.60%	421	20.00%	325		15.50%
5. Somewhat agree	487	23.20%		548	26.10%	567	27.00%	368	17.50%	263		12.50%
6. Agree	257	12.20%		304	14.50%	323	15.40%	220	10.50%	140		6.70%
7. Strongly agree	199	9.50%		230	10.90%	283	13.50%	174	8.30%	110		5.20%

**Table 3:** PART A: Scores at the MPS-R questionnaire for the whole sample (N = 2103 students). PART B: Scores at the SWBS questionnaire for the whole sample (N = 2103 students).

**PART A**	**Whole Sample**			
	**Mean**	**SD**		
When I am working on something, I cannot relax until it is perfect	5.10	1.33		
I find it difficult to meet others’ expectations of me.	4.14	1.70		
One of my goals is to be perfect in everything I do	4.63	1.71		
Everything that others do must be of top-notch quality	3.35	1.50		
I feel that people are too demanding of me	4.38	1.68		
It makes me uneasy to see an error in my work	5.62	1.25		
I cannot stand to see people close to me make mistakes	3.20	1.59		
The people around me expect me to succeed at everything I do	4.54	1.66		
I do not have to be the best at whatever I am doing	3.50	1.75		
I have high expectations for the people who are important to me	4.66	1.45		
My family expects me to be perfect	3.71	1.87		
I set very high standards for myself	5.49	1.47		
The people who matter to me should never let me down	4.86	1.60		
Success means that I work even harder to please others	3.08	1.86		
SOP (Self-oriented perfectionism)	24.30	5.37	4.86 ^	1.07 ^
OOP (Other-oriented perfectionism)	16.10	4.36	4.03 ^	1.09 ^
SPP (Socially prescribed perfectionism)	19.90	6.23	3.98 ^	1.56 ^
MPS-R	60.30	11.90	4.31 ^	0.85 ^
**PART B**	**Whole Sample**			
	**Mean**	**SD**		
1 I don’t find much satisfaction in private prayer with God	2.95	1.8		
2 I don’t know who I am, where I came from, or where I’m going	4.28	1.58		
3 I believe that God loves me and cares about me	3.14	1.76		
4 I feel that life is a positive experience	4.39	1.23		
5 I believe that God is impersonal and not interested in my daily situations	3.3	1.77		
6 I feel unsettled about my future	2.84	1.48		
7 I have a personally meaningful relationship with God	2.55	1.64		
8 I feel very fulfilled and satisfied with life	3.55	1.24		
9 I don’t get much personal strength and support from my God	2.88	1.72		
10 I feel a sense of well-being about the direction my life is headed in	3.61	1.25		
11 I believe that God is concerned about my problems	2.73	1.64		
12 I don’t enjoy much about life	4.71	1.38		
13 I don’t have a personally satisfying relationship with God	2.93	1.79		
14 I feel good about my future	4.02	1.28		
15 My relationship with God helps me not to feel lonely	2.63	1.67		
16 I feel that life is full of conflict and unhappiness	4.07	1.38		
17 I feel most fulfilled when I’m in close communion with God	2.5	1.61		
18 Life doesn’t have much meaning	5.03	1.31		
19 My relation with God contributes to my sense of well-being	2.58	1.65		
20 I believe there is some real purpose for my life	4.12	1.46		
RWB	28.2	15.2	2.82 ^	1.52 ^
EWB	40.6	9.75	4.06 ^	0.98 ^
SWBS	68.8	20.8	3.44 ^	1.04 ^

Legend: SD = Standard Deviation; ^ = Weighted value for the number of items.

**Table 4:** Spearman’s correlation analysis between dimensions of perfectionism and the components of spiritual well-being.

	1 I Don’t Find Much Satisfaction in Private Prayer with God	2 I Don’t Know Who I Am, Where I Came from, or Where I’m Going	3 I Believe That God Loves Me and Cares About Me	4 I Feel That Life Is a Positive Experience	5 I Believe That God Is Impersonal and Not Interested in My Daily Situations	6 I Feel Unsettled About My Future	7 I Have a Personally Meaningful Relationship with God	8 I Feel Very Fulfilled and Satisfied with Life	9 I Don’t Get Much Personal Strength and Support from my God	10 I Feel a Sense of Well-Being About the Direction My Life Is Headed in	11 I Believe That God Is Concerned About My Problems	12 I Don’t Enjoy Much About Life	13 I Don’t Have a Personally Satisfying Relationship with God	14 I Feel Good About My Future	15 My Relationship with God Helps Me Not to Feel Lonely	16 I Feel That Life Is Full of Conflict and Unhappiness	17 I Feel Most Fulfilled When I’m in Close Communion with God	18 Life Doesn’t Have Much Meaning	19 My Relation with God Contributes to My Sense of Well-Being	20 I Believe There Is Some Real Purpose for My Life
When I am working on something I cannot relax until it is perfect	−0.021	−0.043 *	0.002	−0.044 *	−0.031	−0.107 ***	0.010	−0.051 *	−0.005	−0.041	0.015	−0.076 ***	−0.010	−0.026	−0.009	−0.095 ***	−0.004	−0.014	0.005	0.044 *
I find it difficult to meet others’expectations of me	−0.094 ***	−0.324 ***	−0.120 ***	−0.294 ***	−0.142 ***	−0.397 ***	−0.071 **	−0.405 ***	−0.122 ***	−0.376 ***	−0.130 ***	−0.319 ***	−0.109 ***	−0.366 ***	−0.090 ***	−0.314 ***	−0.076 ***	−0.260 ***	−0.083 ***	−0.211 ***
One of my goals is to be perfect in every thing I do	0.030	−0.057 **	0.035	−0.056 **	−0.006	−0.111 ***	0.046 *	−0.037	0.014	−0.047 *	0.034	−0.098 ***	0.031	−0.028	0.040	−0.126 ***	0.040	−0.048 *	0.051 *	0.052 *
Every thing that others do must be of top notch quality	0.079 ***	−0.055*	0.055*	−0.034	0.007	−0.014	0.117 ***	0.027	0.082 ***	−0.006	0.077 ***	−0.075 ***	0.085 ***	0.006	0.102 ***	−0.104 ***	0.110 ***	−0.059 **	0.107 ***	0.034
I feel that people are too demanding of me	−0.030	−0.243 ***	−0.059 **	−0.228 ***	−0.093 ***	−0.302 ***	−0.011	−0.284 ***	−0.050 *	−0.281 ***	−0.055 *	−0.254 ***	−0.046 *	−0.247 ***	−0.039	−0.249 ***	−0.033	−0.196 ***	−0.039	−0.105 ***
It makes me uneasy to see an error in my work	−0.057 **	−0.097 ***	−0.074 ***	−0.069 **	−0.095 ***	−0.122 ***	−0.050 *	−0.093 ***	−0.055 *	−0.090 ***	−0.069 **	−0.089 ***	−0.063 **	−0.065 **	−0.069 **	−0.161 ***	−0.059 **	−0.058 **	−0.062 **	−0.001
I cannot stand to see people close to me make mistakes	0.072 ***	−0.037	0.035	−0.018	0.019	0.012	0.061 **	0.052 *	0.058 **	0.025	0.069 **	−0.034	0.051 *	0.021	0.084 ***	−0.069 **	0.082 ***	−0.016	0.081 ***	0.057 **
The people around me expect me to succeed at everything I do	0.004	−0.160 ***	−0.007	−0.108 ***	−0.052 *	−0.180 ***	0.014	−0.130 ***	−0.023	−0.136 ***	−0.022	−0.152 ***	−0.022	−0.130 ***	−0.004	−0.171 ***	0.018	−0.090 ***	0.019	−0.023
I do not have to be the best at whatever I am doing.	−0.002	−0.090 ***	−0.034	−0.129 ***	−0.052 *	−0.055 *	−0.025	−0.102 ***	−0.002	−0.106 ***	−0.020	−0.124 ***	−0.016	−0.089 ***	−0.030	−0.115 ***	−0.010	−0.101 ***	−0.009	−0.020
I have high expectations for the people who are important to me	0.058 **	0.065 **	0.065 **	0.085 ***	0.053 *	0.002	0.058 **	0.087 ***	0.077 ***	0.093 ***	0.060 **	0.063 **	0.070 **	0.122 ***	0.059 **	0.029	0.063 **	0.079 ***	0.066 **	0.106 ***
My family expects me to be perfect	0.005	−0.167 ***	−0.021	−0.132 ***	−0.078 ***	−0.148 ***	0.010	−0.160 ***	−0.028	−0.143 ***	−0.037	−0.186 ***	−0.025	−0.107 ***	−0.010	−0.157 ***	−0.003	−0.126 ***	−0.001	−0.055 *
I set very high standards for myself	−0.090 ***	−0.056 *	−0.068 **	−0.028	−0.066 **	−0.081 ***	−0.066 **	−0.011	−0.082 ***	−0.021	−0.076 ***	−0.037	−0.079 ***	0.016	−0.082 ***	−0.053 *	−0.079 ***	−0.026	−0.085 ***	0.028
The people who matter to me should never let me down	0.039	−0.011	0.032	−0.011	0.032	−0.028	0.048*	0.011	0.030	0.000	0.048 *	0.004	0.047 *	0.046 *	0.039	−0.067 **	0.037	0.027	0.041	0.078 ***
Success means that I work even harder to please others	0.000	−0.216 ***	−0.023	−0.200 ***	−0.046 *	−0.128 ***	0.022	−0.164 ***	−0.006	−0.175 ***	−0.022	−0.241 ***	−0.007	−0.178 ***	0.017	−0.226 ***	0.014	−0.216 ***	0.006	−0.082 ***

Legend: * *p* < 0.05, ** *p* < 0.01, *** *p* < 0.001.

**Table 5:** Multiple linear regression analysis explaining differences in perfectionism (MPS-R).

Predictor	β	SE	t	*p*
Constant ^a^	73.219	2.070	35.370	<0.001 *
RWB	0.067	0.024	2.754	0.006 *
EWB	−0.369	0.028	−13.117	<0.001 *
Sex				
Female-Male	1.662	0.597	2.785	0.005 *
Age				
Young adults-Young	−2.825	0.645	−4.379	<0.001 *
Middle Age-Young	−3.929	1.491	−2.635	0.008 *
Religion				
Other religion-Catholic	1.296	1.061	1.222	0.222
Atheist/Agnostic-Catholic	0.398	0.773	0.515	0.607
Indifferent-Catholic	−0.971	0.866	−1.121	0.262
Area of study				
Science-Health science	−1.182	1.185	−0.997	0.319
Humanities-Health science	1.141	0.922	1.237	0.216
Engineering-Health science	1.216	1.005	1.210	0.226
Others-Health science	0.761	1.614	0.471	0.637
Level of study				
Master’s degree-Bachelor’s degree	1.299	0.715	1.815	0.070
Location				
Centre-North	−1.285	0.893	−1.439	0.150
South-North	0.653	1.276	0.512	0.609
Islands-North	0.948	0.867	1.093	0.274
Private University				
Yes-No	0.708	1.394	0.508	0.612
Off site				
Yes-No	−3.093	2.825	−1.095	0.274
Scholarship/place in College of Merit				
Yes-No	2.521	2.666	0.946	0.344

Legend: β = standardized regression coefficient; SE = standard error of the coefficient; t = t-statistic (ratio of coefficient to its standard error); ^a^ = reference level; * = Statistically significant.

## Data Availability

The data supporting the conclusions of this article may be made available upon reasonable request to qualified researchers by contacting the corresponding authors.

## Supplementary Materials

The following supporting information can be downloaded at: https://www.mdpi.com/article/10.3390/ejihpe15100211/s1, Table S1. Items of the Multidimensional Perfectionism Scale—Revised (MPS-R).

## Abbreviations

The following abbreviations are used in this manuscript:

MPS-R	Multidimensional Perfectionism Scale—Revised
SOP	Self-Oriented Perfectionism
OOP	Other-Oriented Perfectionism
SPP	Socially Prescribed Perfectionism
SWBS	Spiritual Well-Being Scale
EWB	Existential Well-Being
RWB	Religious Well-Being
SD	Standard Deviation
STROBE	Strengthening the Reporting of Observational Studies in Epidemiology

## Appendix A

**Table A1:** PART A: Scores on the MPS-R questionnaire divided by sex (N = 2103 students). PART B: Scores on the SWBS questionnaire divided by sex (N = 2103 students).

**PART A**	**Whole Sample**		**Man**		**Woman**		
	**Mean**	**SD**	**Mean**	**SD**	**Mean**	**SD**	***p*-Value ^$^**
When I am working on something, I cannot relax until it is perfect	5.10	1.33	5.00	1.29	5.13	1.35	0.012 *
I find it difficult to meet others’ expectations of me.	4.14	1.70	3.91	1.74	4.22	1.69	<0.001 *
One of my goals is to be perfect in everything I do	4.63	1.71	4.46	1.78	4.69	1.68	0.010 *
Everything that others do must be of top-notch quality	3.35	1.50	3.50	1.55	3.31	1.49	0.028 *
I feel that people are too demanding of me	4.38	1.68	4.04	1.73	4.48	1.65	<0.001 *
It makes me uneasy to see an error in my work	5.62	1.25	5.50	1.29	5.66	1.23	0.016 *
I cannot stand to see people close to me make mistakes	3.20	1.59	3.35	1.66	3.16	1.57	0.040 *
The people around me expect me to succeed at everything I do	4.54	1.66	4.30	1.67	4.62	1.66	<0.001 *
I do not have to be the best at whatever I am doing	3.50	1.75	3.57	1.80	3.48	1.74	0.343
I have high expectations for the people who are important to me	4.66	1.45	4.76	1.42	4.63	1.46	0.161
My family expects me to be perfect	3.71	1.87	3.60	1.87	3.75	1.87	0.155
I set very high standards for myself	5.49	1.47	5.36	1.56	5.53	1.45	0.058
The people who matter to me should never let me down	4.86	1.60	4.87	1.60	4.85	1.60	0.804
Success means that I work even harder to please others	3.08	1.86	2.89	1.82	3.13	1.86	0.010 *
SOP (Self-oriented perfectionism)	24.30	5.37	23.90	5.39	24.50	5.36	0.020 *
OOP (Other-oriented perfectionism)	16.10	4.36	16.50	4.49	16.00	4.31	0.024 *
SPP (Socially prescribed perfectionism)	19.90	6.23	18.70	6.10	20.20	6.24	<0.001 *
MPS-R	60.30	11.90	59.10	11.70	60.60	12.00	0.010 *
**PART B**	**Whole Sample**		**Man**		**Woman**		
	**Mean**	**SD**	**Mean**	**SD**	**Mean**	**SD**	***p*-Value ^$^**
1 I don’t find much satisfaction in private prayer with God	2.95	1.8	2.78	1.87	3	1.78	0.005 *
2 I don’t know who I am, where I came from, or where I’m going	4.28	1.58	4.28	1.69	4.28	1.55	0.529
3 I believe that God loves me and cares about me	3.14	1.76	2.91	1.82	3.21	1.73	<0.001 *
4 I feel that life is a positive experience	4.39	1.23	4.39	1.3	4.39	1.21	0.564
5 I believe that God is impersonal and not interested in my daily situations	3.3	1.77	3.03	1.83	3.38	1.75	<0.001 *
6 I feel unsettled about my future	2.84	1.48	2.99	1.51	2.79	1.48	0.006 *
7 I have a personally meaningful relationship with God	2.55	1.64	2.44	1.67	2.59	1.63	0.033 *
8 I feel very fulfilled and satisfied with life	3.55	1.24	3.54	1.27	3.55	1.23	0.940
9 I don’t get much personal strength and support from my God	2.88	1.72	2.72	1.8	2.93	1.69	0.005 *
10 I feel a sense of well-being about the direction my life is headed in	3.61	1.25	3.63	1.28	3.6	1.24	0.498
11 I believe that God is concerned about my problems	2.73	1.64	2.52	1.66	2.8	1.63	<0.001 *
12 I don’t enjoy much about life	4.71	1.38	4.64	1.46	4.73	1.35	0.383
13 I don’t have a personally satisfying relationship with God	2.93	1.79	2.85	1.86	2.96	1.78	0.119
14 I feel good about my future	4.02	1.28	4.04	1.32	4.02	1.27	0.392
15 My relationship with God helps me not to feel lonely	2.63	1.67	2.48	1.7	2.68	1.65	0.006 *
16 I feel that life is full of conflict and unhappiness	4.07	1.38	3.84	1.44	4.14	1.35	<0.001 *
17 I feel most fulfilled when I’m in close communion with God	2.5	1.61	2.4	1.62	2.53	1.6	0.058
18 Life doesn’t have much meaning	5.03	1.31	4.87	1.47	5.09	1.25	0.022 *
19 My relation with God contributes to my sense of well-being	2.58	1.65	2.49	1.68	2.61	1.64	0.068
20 I believe there is some real purpose for my life	4.12	1.46	3.89	1.59	4.19	1.41	<0.001 *
RWB	28.2	15.2	26.6	15.5	28.7	15	0.002 *
EWB	40.6	9.75	40.1	10.2	40.8	9.6	0.409
SWBS	68.8	20.8	66.7	21.9	69.4	20.4	0.008 *

^$^ Mann Witney U-test; * Statistically significant.
