# Supplementary material for: The Impact of Spiritual Well-Being on Multidimensional Perfectionism in University Students: A Nationwide Survey
Source: Eur J Investig Health Psychol Educ. 2025 Oct 15;15(10):211. doi: 10.3390/ejihpe15100211 (PMC12564579; doi:10.3390/ejihpe15100211)
Supplement: Supplementary file 1 [file ejihpe-15-00211-s001.zip › ejihpe-3887416-supplementary.pdf]

Table S1. Items of the Multidimensional Perfectionism Scale – Revised (MPS-R)

1. When I am working on something, I cannot relax until it is perfect
2. I find it difficult to meet others' expectations of me
3. One of my goals is to be perfect in everything I do
4. Everything that others do must be of top-notch quality
5. I feel that people are too demanding of me
6. It makes me uneasy to see an error in my work
7. I cannot stand to see people close to me make mistakes
8 The people around me expect me to succeed at everything I do
9. I do not have to be the best at whatever I am doing
10. I have high expectations for the people who are important to me
11. My family expects me to be perfect
12. I set very high standards for myself
13. The people who matter to me should never let me down
14. Success means that I work even harder to please others
